# Obesity results in lower integrin expression in women with polycystic ovarian syndrome during the window of implantation

**DOI:** 10.3389/fendo.2025.1590716

**Published:** 2025-10-21

**Authors:** Fazilah Abdul Hamid, Mohd Helmy Mokhtar, Abdul Kadir Abdul Karim, Mohd Faizal Ahmad, Nor Haslinda Abd Aziz, Azantee Yazmie Abdul Wahab, Muhammad Azrai Abu

**Affiliations:** ^1^ Department of Physiology, Faculty of Medicine, Universiti Kebangsaan Malaysia, Kuala Lumpur, Malaysia; ^2^ Advance Reproductive Centre, Hospital Canselor Tunku Muhriz, Kuala Lumpur, Malaysia; ^3^ Department of Obstetrics and Gynaecology, Faculty of Medicine, Universiti Kebangsaan Malaysia, Kuala Lumpur, Malaysia; ^4^ Department of Biomedical Science, Kulliyah of Allied Health Sciences, International Islamic University Malaysia, Kuantan, Pahang, Malaysia

**Keywords:** gene expression, integrin, implantation window, obese, polycystic ovary syndrome, obesity

## Abstract

**Objective:**

Polycystic ovary syndrome (PCOS) is a common endocrine disorder that is characterized by hormonal imbalances and ovarian dysfunction. Obesity is also a prevalent issue that has been linked to the development of PCOS. The present study aimed to investigate the gene expression of αvβ3 integrin, mucin-1, and E-cadherin in obese and non-obese women with and without PCOS.

**Methods:**

This prospective study was undertaken at the Advanced Reproductive Centre at Hospital Canselor Tuanku Muhriz (Universiti Kebangsaan Malaysia) from January 2019 to June 2021. A total of 40 women were recruited for the study and divided equally (n = 10) into four groups, namely, i) control with normal body weight, ii) control obese, iii) PCOS with normal body weight, and iv) PCOS obese. An endometrial tissue sample was collected after 10 days of daily oral micronized progesterone (Utrogestan 200 mg) in the PCOS group. In the fertile or control group, midsecretory phase endometrial biopsy was performed following 7 days post-ovulation. Then, total RNA was isolated from the endometrial tissue. Gene expression was analyzed using RT-qPCR.

**Results:**

The results showed that the mRNA expression of αVβ3-integrin was significantly decreased in the PCOS obesity group compared to the PCOS normal body weight group and the control normal body weight group. No significant differences were observed in mucin-1 and E-cadherin expression between the groups.

**Conclusion:**

αvβ3 integrin plays an important role in the development of the window of implantation in obese PCOS individuals. Further research is needed to confirm these results and to identify the potential mechanisms underlying this association.

**Clinical trial registration:**

ClinicalTrial.gov, identifier NCT04175002.

## Introduction

Polycystic ovary syndrome (PCOS) is a common endocrine disorder that affects up to 15% of women of reproductive age. It is characterized by hormonal imbalances, including elevated levels of androgens and insulin, and ovarian dysfunction (1). The exact cause of PCOS is unknown, but it is thought to be influenced by a combination of genetic and environmental factors (2). One of these factors is obesity, which has been linked to the development of PCOS (3–5). Obesity is defined as having a body mass index (BMI) of 30 or higher and is associated with a range of health problems, including diabetes, cardiovascular diseases, and certain cancers (6, 7).

Several studies have suggested that obesity may contribute to the development of PCOS through various mechanisms, including insulin resistance and inflammation (1, 3, 8, 9). Insulin resistance, which is characterized by a reduced sensitivity of cells to insulin, is a common feature of obesity and is thought to play a role in the development of PCOS (8). Inflammation, which is the body’s response to tissue damage or infection, has also been implicated in the pathogenesis of PCOS (9, 10).

Cell adhesion molecules are molecules that are found on the surface of cells that regulate cell–cell adhesion and contact between cells and the extracellular matrix (ECM). Based on the structures and functional similarities, it was divided into five groups, which are integrins, cadherins, mucins, selectins, and the immunoglobulin superfamily (11). αvβ3 integrin, mucin-1, and E-cadherin are proteins that are involved in various biological processes, including cell adhesion, communication, and signaling (12, 13). Previous studies have shown that these proteins may be differentially expressed in women with PCOS, but the results have been inconsistent (14, 15). It is a well-known fact that cell adhesion molecules such as integrin αvβ3, MUC-1, and E-cadherin play an important role in endometrial receptivity, which is a term to describe the endometrium’s start during the window of implantation (WOI) (16–18). MUC-1 is a transmembrane protein that has a few functions, such as cellular lubrication, epithelial protection, and anti-adhesion activity; it has been reported to be highly expressed in the endometrium and is the first molecule on the surface of the uterus where the embryo is being implanted, which suggests that an imbalance in this molecule may affect the embryo implantation adhesion process (12, 19). The same can be said for other cell adhesion molecules, such as integrin αvβ3 and E-cadherin. There is increasing evidence suggesting that obesity and PCOS negatively affect endometrial receptivity (3). Few studies have reported conflicting results regarding the levels of these cell adhesion molecules; for example, some have reported lower expression of integrin αvβ3 in women with PCOS, some have reported high expression of MUC-1 in PCOS women, and some have found no significant relationship between MUC-1 and PCOS (15–19). There is also a limited study involving the associations of these molecules with obesity (15). The present study aimed to investigate the gene expression of αvβ3 integrin, mucin-1, and E-cadherin in obese and non-obese women with and without PCOS.

## Methods

### Study design

This prospective study was undertaken at the Advanced Reproductive Centre at Hospital Canselor Tuanku Muhriz (Universiti Kebangsaan Malaysia) from January 2019 to June 2021. Ethical approval was obtained from the Research and Ethics Committee of Universiti Kebangsaan Malaysia (UKM.FPR.SPI 800-2/28/6) and funded by the National Fundamental Research Grant Scheme (FRGS/1/2018/SKK08/UKM/03/2).

### Sample collection

A purposive sampling to obtain endometrial tissue samples from 10 consented subjects in each of the study groups was conducted on women attending the Advanced Reproductive Centre. The study groups consisted of the following:

1. normal-weight controls with BMI less than 27 (C-NW),
2. obese controls with BMI greater than 27 (C-OB),
3. PCOS women with normal weight with BMI less than 27 (P-NW), and
4. obese PCOS women with BMI greater than 27 (P-OB).

Diagnosis of PCOS was confirmed by the presence of at least two out of the three diagnostic criteria, namely, loss of ovarian function (oligo-anovulation), clinical or biochemical androgen excess, and evidence of polycystic ovaries on ultrasound. The recruited PCOS control population consisted of consented healthy women with confirmed fertility (with at least one child), a normal level of basic reproductive hormones, and a regular menstrual cycle interval. Obesity was defined as having a BMI of 27 kg/m^2^ or higher.

Additionally, women attending the Advanced Reproductive Centre who were smoking, had hormonal treatment for anovulation for at least 3 months prior to sample collection, had any pregnancy or lactation during the previous 12 months, had other systemic diseases (including endocrine and eating disorders and uterine or ovarian diseases), on any regular medication (such as insulin sensitizers, hormones, herbal substance, statins, or corticoids for at least 3 months before sample collection), and had a history of intrauterine device replacement were excluded from the sampling population.

Endometrial tissue sample collection was taken during the WOI. For the PCOS groups, the WOI was marked by the completion of daily oral micronized progesterone (Utrogestan 200 mg) therapy for 10 days. For the control group, the WOI was marked by 7 days post-confirmation of ovulation via a urine ovulation test.

### RNA extraction

Total RNA was isolated from endometrial tissue with an RNeasy Plus Mini Kit (Qiagen, Hilden, Germany) following the manufacturer’s instructions. RNA was eluted with 30 µL of RNAase–DNAse-free water, and then its concentration and quality were measured. The total RNA was then reverse transcribed into cDNA using the iScript™ cDNA Synthesis Kit (Bio-Rad, California, United States).

### Reverse transcriptase quantitative polymerase chain reaction

The expression of αvβ3 integrin, mucin-1, and E-cadherin genes was compared to that of glyceraldehyde 3-phosphate dehydrogenase (GAPDH) as the reference gene. Primers designed for the target and reference genes are depicted in **Table 1**. SsoAdvanced™ Universal SYBR^®^ Green Supermix (Bio-Rad) was utilized in the qPCR master mix, and the thermal cycler applied was an iCycler iQ™ Real-Time PCR Detection System (Bio-Rad). The cycling conditions included denaturation at 98°C for 30 s, 40 cycles of 95°C for 15 s, and 60°C for 30 s. The melting curve was set at 65°C to 95°C for 5 s with an increment of 0.5°C.

**Table 1:** Primer design for RT-qPCR target genes and reference genes.

Gene	Primer	BP	GC content
αVβ3 integrin	5′-AAACTCCTCATCACCATCCAC-3′ 5′-CGGTACGTGATATTGGTGAAGG-3′	208	47.6% 50.0%
Mucin-1	5′-GAGTACCCCACCTACCACA-3′ 5′-GCCACCATTACCTGCAGAA-3′	203	57.9% 52.6%
E-cadherin	5′-GTCTGTCATGGAAGGTGCTC-3′ 5′-CTGAGGATGGTGTAAGCGATG-3′	314	55.0% 52.4%
GAPDH	5′-TCA AGG CTG AGA ACG GGA AG-3′ 5′-TCG CCC CAC TTG ATT TTG GA-3′	88	55.0% 50.0%

BP, base pair; GC, Guaninie cytosine content.

### Data analysis

The gene expression levels were calculated using the 2−ΔΔCt method and were normalized to the reference gene (GAPDH). The data were analyzed using the Student’s t-test or the Mann–Whitney U test, as appropriate. A p-value of <0.05 was considered statistically significant.

## Results

### Sociodemographic data

A total of 40 women aged between 29 and 41 years were included in this study. The participants were categorized into four groups according to their diagnosis of PCOS and BMI measurements. The mean ± standard deviation of the age and BMI of study participants according to the study groups is shown in **Table 2**.

**Table 2:** Demographic characteristics of the study participants.

Study groups	Age (years)	P-value	BMI (kg/m^2^)	P-value
PCOS with normal body weight (n = 10)	35.80 ± 2.90		23.05 ± 1.46	
Obese PCOS (n = 10)	37.00 ± 2.83	0.412	31.25 ± 1.62	0.044*
Control with normal body weight (n = 10)	37.10 ± 2.77		24.40 ± 1.94	
Obese control (n = 10)	33.20 ± 2.74	0.021*	32.46 ± 1.53	<0.001*

BMI, body mass index; PCOS, polycystic ovary syndrome.

* show p value <0.05.

### Obesity results in the downregulation of αVβ3 integrin gene expression in PCOS women

**Figure 1:**
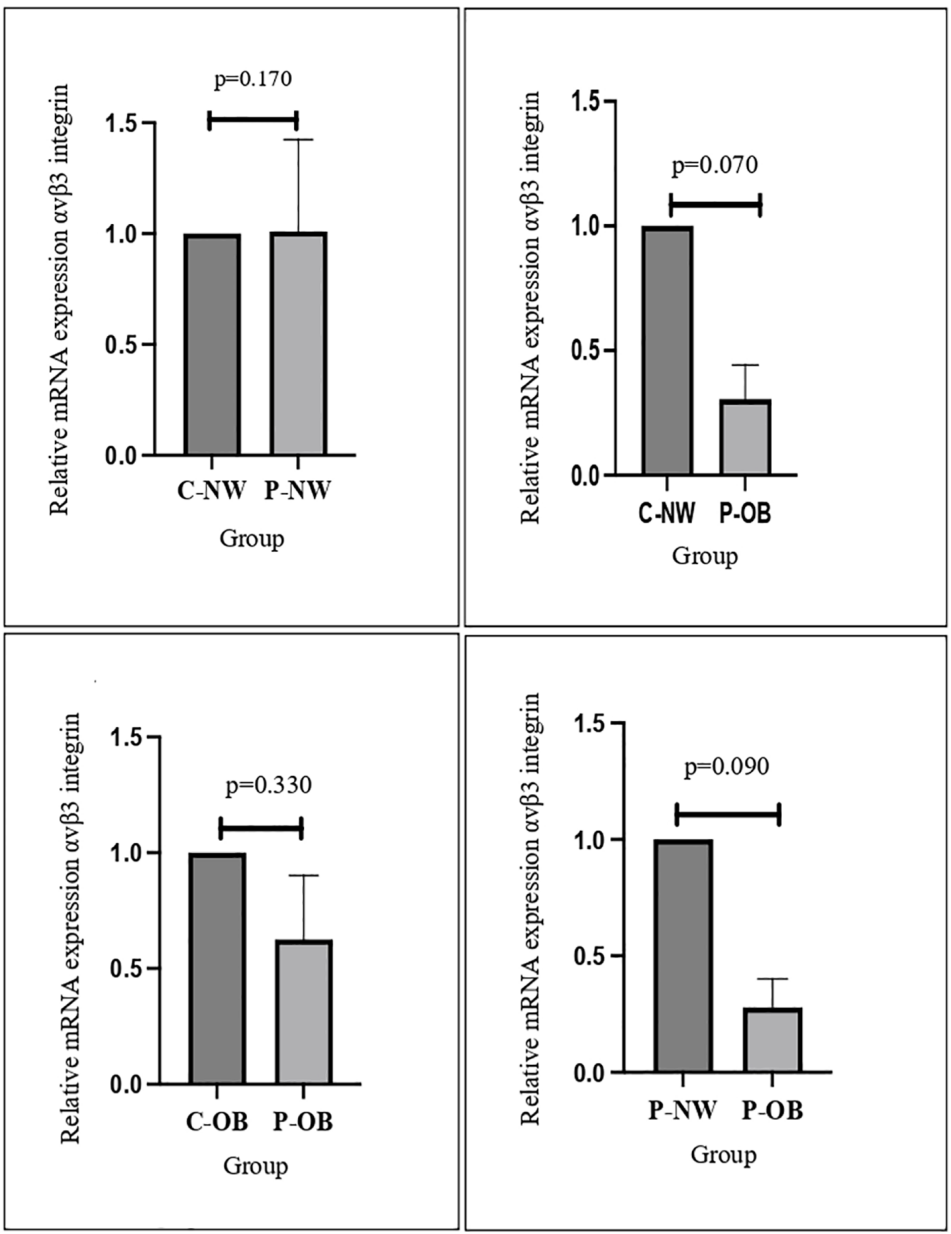
The results of qPCR analysis showed that mRNA αvβ3 integrin decreased significantly in the obese PCOS group compared to the normal-weight control group.

This study demonstrated that αvβ3 integrin expression was significantly lower in obese PCOS women compared to both non-obese PCOS women and fertile women with normal body weight. However, no significant difference in integrin expression was observed between non-obese PCOS women and fertile controls (**Figure 1**). These findings suggest that obesity, rather than PCOS itself, is the main factor driving the downregulation of αvβ3 integrin expression following progesterone therapy. In contrast, among healthy fertile women (non-PCOS), obesity did not significantly influence integrin levels. Furthermore, the expression of MUC-1 and E-cadherin remained unchanged across all groups, indicating that these markers were not affected by either PCOS status or obesity.

## Discussion

The present study aimed to investigate the gene expression of αvβ3 integrin, mucin-1, and E-cadherin in obese and non-obese women with and without PCOS. Our results showed that αvβ3 integrin expression was significantly lower in obese women in both the PCOS and non-PCOS groups compared to their non-obese counterparts. No significant differences were observed in mucin-1 and E-cadherin expression between the groups.

αvβ3 integrin is a protein that is expressed on the surface of cells and is involved in various biological processes, including cell adhesion, migration, and signaling (20). Previous studies have suggested that αvβ3 integrin may play a role in the development of PCOS. For example, one study reported that αvβ3 integrin expression was increased in theca cells (steroidogenic cells in the ovaries) from women with PCOS compared to controls. Another study found that αvβ3 integrin expression was reduced in the ovarian tissue of women with PCOS compared to controls (17, 21, 22). The results of the present study are in line with the latter finding and suggest that αvβ3 integrin may be downregulated in obese women with PCOS.

It is not clear why αvβ3 integrin expression was lower in obese women with PCOS in the present study. One possible explanation is that obesity may affect the expression of this protein through various mechanisms, including insulin resistance and inflammation. Insulin resistance, which is a common feature of obesity, has been linked to the development of PCOS and may affect the expression of αvβ3 integrin (3, 8, 14). Inflammation, which is also associated with obesity, has been implicated in the pathogenesis of PCOS and may also play a role in the regulation of αvβ3 integrin expression (3, 23). Further research is needed to confirm these results and to identify the potential mechanisms underlying this association.

**Figure 2:**
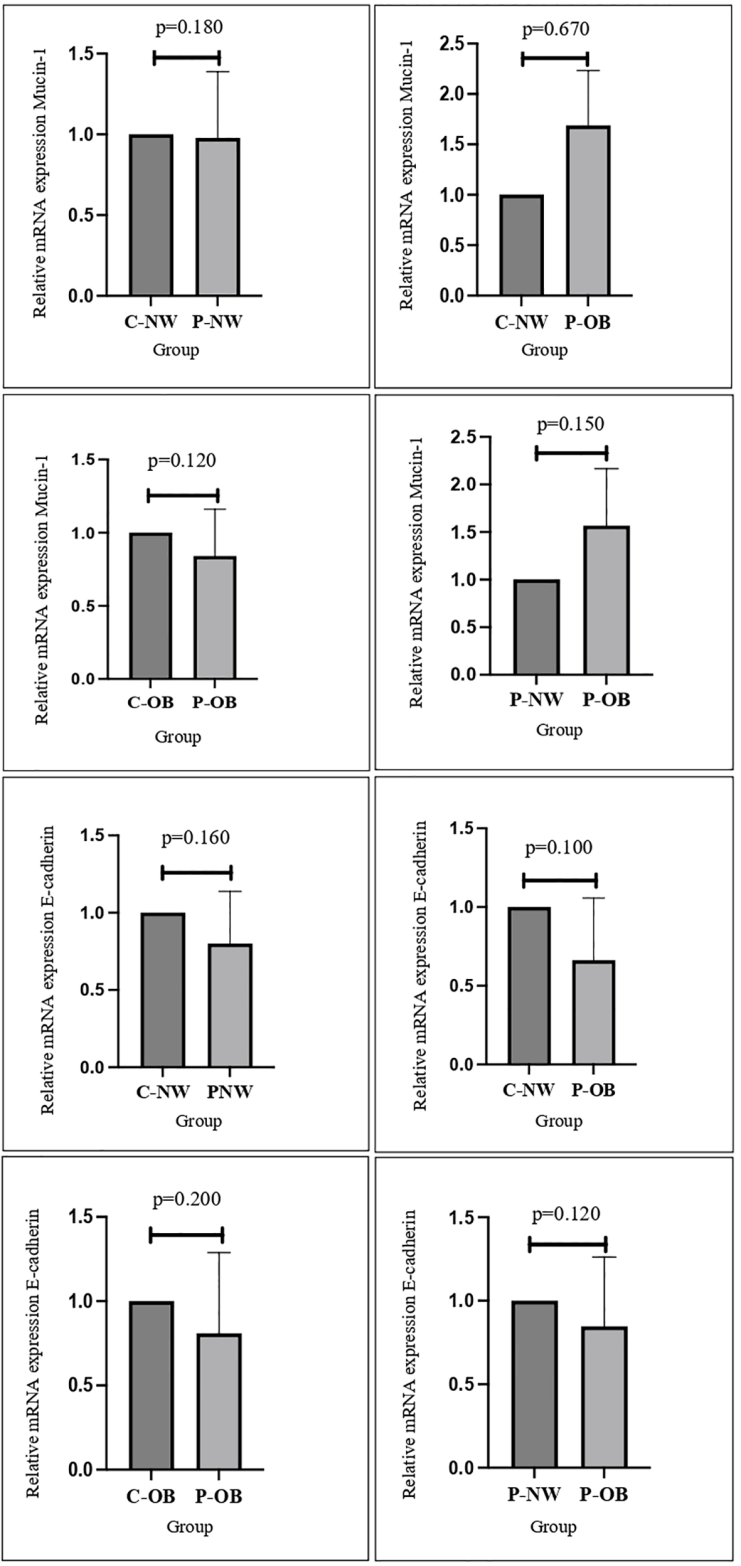
No significant differences were found in the mRNA expression and protein distribution of E-cadherin and mucin-1 in all study groups.

As shown in **Figure 2**, there is no significant difference in the expression of E-cadherin and MUC-1 between obese and non-obese women with or without PCOS. This is in concordance with a study performed by Margaret et al. that found no difference between MUC-1 expression in women with or without PCOS (24). E-cadherin and MUC-1 have been known to have an important role in endometrial receptivity (17). A study performed by Budihastuti et al. showed that higher expression of MUC-1 is related to a reduction in endometrial receptivity, and women with PCOS have been reported to have higher expression of MUC-1 (19). Regarding obesity and MUC-1 expression, a study performed by Wu et al. reported that BMI was not significantly related to expression of endometrial MUC-1, which is in line with the results of this study (15). While there is increasing evidence suggesting that MUC-1 and E-cadherin are some of the biomarkers that are deregulated in the endometrium of PCOS patients, there are limited studies that associate weight and patients’ BMI with endometrial receptivity markers (17, 19, 25). A study performed by Bergant et al. reported that there are no significant changes in the biomarker expression of genes involved in endometrial receptivity in association with the BMI of patients during the WOI, which agrees with the results of this study (26).

There is a limitation in our study. The total number of participants included was relatively small. The relatively small sample size in each of the four groups may result in small or subtle differences. The small number of participants may be due to the highly specific criteria needed in this study, and when all the inclusion and exclusion criteria were met, not many women remained to be included in this study. Nevertheless, a significant difference in αvβ3 integrin expression between the non-obese with PCOS group and the obese with PCOS group suggests that the difference is likely to be of significant biological relevance.

In conclusion, the present study showed that αvβ3 integrin expression was significantly lower in obese women with and without PCOS compared to their non-obese counterparts. These findings suggest that αvβ3 integrin may play a role in the development of PCOS in obese individuals. Further research is needed to confirm these results and to identify the potential mechanisms underlying this association.

## Data Availability

The original contributions presented in the study are included in the article/supplementary material, further inquiries can be directed to the corresponding author.
